# Oral *Porphyromonas gingivalis* Infections Increase the Risk of Alzheimer’s Disease: A Review

**DOI:** 10.3290/j.ohpd.b3818045

**Published:** 2023-01-18

**Authors:** Yuwen Fu, Xin Xu, Yu Zhang, Peng Yue, Yuxin Fan, Meixiao Liu, Jingjing Chen, Aihua Liu, Xiufeng Zhang, Fukai Bao

**Affiliations:** a Bachelor’s Student, Yunnan Province Key Laboratory for Tropical Infectious Diseases in Universities, Kunming Medical University, Kunming, Yunnan, China; College of Stomatology – Kunming Medical University, Kunming, Yunnan, China. Conducted the database search and screening drafted the manuscript.; b Master’s Student, Yunnan Province Key Laboratory for Tropical Infectious Diseases in Universities, Kunming Medical University, Kunming, Yunnan, China. Conducted the database search and screening.; c Professor, Yunnan Province Key Laboratory for Tropical Infectious Diseases in Universities, Kunming Medical University, Kunming, Yunnan, China. Conceived and designed the study, revised and approved the manuscript.; d Associate Professor, College of Forensic Medicine – Kunming Medical University, Kunming, Yunnan, China. Conceived and designed the study.; e Professor, Yunnan Province Key Laboratory for Tropical Infectious Diseases in Universities, Kunming Medical University, Kunming, Yunnan, China. Conceived and designed the study, revised and approved the manuscript.

**Keywords:** Alzheimer’s disease, neurodegenerative disease, periodontal disease, *Porphyromonas gingivalis* (*P. gingivalis*)

## Abstract

Periodontal disease (PD) and Alzheimer’s disease (AD) are inflammatory diseases affecting the adult population of the world. PD is mainly caused by infection with *Porphyromonas gingivalis* (*P. gingivalis*) and by the synergistic action of various microorganisms. These microorganisms penetrate into the subgingival tissue and cause bacteremia, leading to disruption of the homeostasis of the internal environment of the body. Virulence factors known as gingipains, which are cysteine proteases and other toxins, including fimbria and lipopolysaccharides (LPS), are strongly associated with periodontitis and other systemic inflammation. PD has a known polymicrobial aetiology, and patients who eventually develop sporadic AD tend to have recurrent infections before a clinical diagnosis of dementia. AD, the most common neurodegenerative disease, is characterised by poor memory and specific hallmark proteins. An increasing number of studies have shown that periodontal pathogens are increasingly associated with this form of dementia. Many articles have shown that *P. gingivalis* infections directly increase the risk of PD and may indirectly lead to the development of AD. However, these links and probable pathogenesis remain to be explored. The aim of this review was to explore whether *P. gingivalis* periodontal infection is associated with AD and to provide possible mechanisms of association.

Periodontitis is highly prevalent in humans, affecting nearly 50% of the population around the world. The cause of multiple microbial infections of chronic periodontitis is the host’s subgingival pathological biota; this triggers hard/soft tissue destruction which progresses with age.^48^ Furthermore, the dysbiotic oral biofilm constituents can affect the function of the brain, which may lead to depression and the development of dementia.^21^ Neurodegenerative diseases are defined as inherited, sporadic and age-related disorders characterised by cognitive decline, especially in memory and learning. These disorders are often associated with problems of mental functioning (dementias) or movement (ataxias).^60^ Periodontitis is closely associated with not only oral diseases but also with general health. *Porphyromonas gingivalis* (*P. gingivalis*) is an asaccharolytic Gram-negative (G-) anaerobic bacillus that produces major virulence factors known as gingipains. Gingipains are cysteine proteases consisting of arginine-gingipain A (RgpA), arginine-gingipain B (RgpB), and lysine-gingipain (Kgp);^27,39^ these are well-known key pathogens of periodontitis, associated with multiple systemic diseases such as diabetes mellitus and Alzheimer’s disease (AD).^28^

Consecutive surveys described biochemical, microbiological and morphological characteristics of oral microorganisms, deepening our understanding of bacterial adherence to the tooth surface.^7,65^ The human oral microbiome is highly diverse, harboring an estimated number of 700 bacterial species.^7,28^ Age-related changes, including the reduction of salivary flow, insufficient oral care, systemic comorbidities, or multiple medications, are likely to impact the composition of the oral microorganisms and the appearance of oral diseases. Changes in diet and oral hygiene habits are accompanied by pathological changes in the oral microorganisms, and periodontal pathogens with greater acid production and acid resistance become dominant.^54,64^ Bacterial species may vary considerably between individuals, even between different sites in the same individual, but remain relatively stable. The oral cavity is a diverse environment with many distinct surfaces, topographies, and microenvironments, so that the microbial population at each ecological site will vary depending on nutrition, pH, host defense factors, and other variables.^27^ Thus, an individual with poor oral hygiene will accumulate a mature biofilm on oral surfaces, which will lead to gingivitis and periodontitis.

Periodontal disease (PD), as a bacterial disease, causes an immune and inflammatory response in the host, involving innate and adaptive signaling mechanisms, including the recruitment of cells from the systemic circulation (plasma cells, macrophages, and T- and β- lymphocytes). These then permeate the gingival soft tissues,^43^ leading to connective-tissue and bone degeneration and necrosis, and finally clinical signs.^27^ The host’s defense failure in the gingival tissues causes the key inflammatory microorganism *P. gingivalis* to colonise the subgingival area and then spread to other, distant organs.^49,54^

AD is a type of chronic progressive encephalopathy. It starts with unnoticeable changes in the brain, then gradually develops into symptoms such as poor memory, cognitive decline and language problems as soon as neurons have been damaged or partly destroyed by specific hallmark proteins.^28^

Traditionally, it has been thought that the physical blood-brain barrier (BBB) and the absence of the lymphatic system in the brain produces an immunologically privileged situation, since low-level molecules are essential for antigen presentation.^57^ It is necessary to emphasise that the BBB can maintain the isolation of the central nervous system and avoid the challenge of abnormal immunity.^26^ Thus, during the course of neurodegenerative disorders, the brain is more dependent on the resident central nervous system, rather than recruitment of peripheral adaptive immune surveillance cells to identify and respond to invading pathogens. While astrocytes and neurons have the ability to respond to infection, microglia are the main guardians of a healthy brain.^61^ The central nervous system is highly protected by the BBB, which consists of pericytes, astrocytes, and microvascular endothelial cells, and the BBB controls the molecules entering and exiting the brain.^29^ However, bacteria can infiltrate the BBB through many mechanisms, including transcellular and paracellular pathways (Fig 1).^19^ Peripheral infection can activate amyloid-beta (Aβ), which activates microglia in the central nervous system, thereby promoting the development of AD neurodegeneration.^26^ This is why many studies have claimed that gingipains and/or LPS of *P. gingivalis* can be found in brain cells.

**Fig 1:**
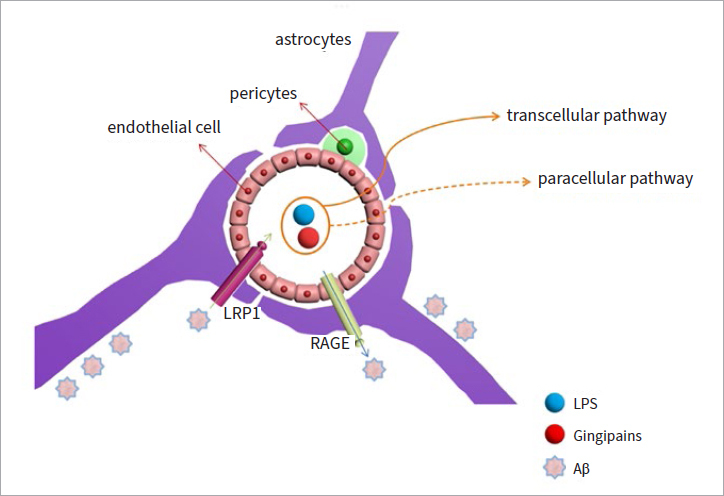
The structure of the BBB and different mechanisms of bacterial toxins. Aβ enters brain tissues through the BBB. The BBB contains three main parts, which are endothelial cells, pericytes, and astrocytes. Bacterial toxins enter the brain through transcellular and paracellular pathways. Receptors for RAGE and LRP1 mediate Aβ entering and exiting brain tissues. Without enough LRP1, Aβ will accumulate and become extracellularly insoluble senile plaques outside the neural cells.

Undoubtedly, human polymorphic genes do affect host susceptibility to disease, but these genes also affect pathogen survival and proliferation.^33^ Thus, *P. gingivalis* is a prime example of a pathogen that survives by adapting to the host’s challenging inflammatory environment.

Amyloid-beta (Aβ) plaque causes senile plaque (SP) and hyperphosphorylated Tau (p-Tau), which binds to neurofibrillary tangles (NFTs), all of which are important diagnostic and neuropathological markers in AD. Intra- and extra-cellularly, in in-vivo and in-vitro animal models, both Aβ and p-Tau can act as toxins.^15,45^ A second factor in AD is infection/inflammation of the brain, in which the key pathogen *P. gingivalis* appears to play an important role in chronic periodontitis. Brain inflammation in the form of activated microglia is the third major histopathological feature, but does not play a role in the neuropathological diagnosis of AD.^15^ Increasing pathological Aβ deposits activate glial cells (astrocytes and microglia), macrophages, and lymphocytes, which in turn release numerous inflammatory mediators such as cytokines, neurotransmitters, chemokines, and reactive oxygen species (ROS). Reactive astrocytes and microglia induce neuronal apoptosis and BBB dysfunction. Furthermore, aging is another major risk factor for AD and is associated with increased glial reactivity, which may increase the brain’s susceptibility to injury and disease.^66^

## *P. gingivalis* Periodontal Infection

Periodontitis is characterised as a low-grade systemic disease with the presence of gingival oedema, congestion, erythema, periodontal pockets, and loss of bone and soft tissue supporting the teeth,^29,30^ as well as release of pro-inflammatory cytokines into the systemic circulation, and an increase of C-reactive protein (CRP).^11^ PD is known to have multiple microbial aetiologies, and patients who eventually develop sporadic AD tend to have recurrent infections before clinical diagnosis of dementia. Multiple clinical,^54^ epidemiological,^3^ and molecular studies^52,57^ have shown that *P. gingivalis* infection-associated chronic inflammation is related to increased risk of AD and other forms of dementia. The main reason may be that *P. gingivalis* and its toxins, such as high levels of LPS within the host, leads to tissue destruction and bone loss by inducing the immune response and production of pro-inflammatory molecules, chemokines, and cytokines, such as interleukin-1β (IL-1β), interleukin-6 (IL-6), tumor necrosis factor-α (TNF-α), and inducible nitric oxide synthase (iNOS), while decreasing the expression of anti-inflammatory mediators, including interleukin-10 (IL-10), arginase-1, and interleukin-4 (IL-4).^10,16,25,33^ These pro-inflammatory cytokines enhance the pool of inflammatory mediators in the brain. That is, active microglia, which are the first brain cells that respond to systemic inflammation, lead to confusion and dementia. *P. gingivalis* lipopolysaccharide is almost the smooth type (called LPS-1),^40,68^ consisting of three regions: lipid A, O polysaccharide, and R polysaccharide. *P. gingivalis* has an additional arsenal that infiltrates into different organs, inhibits phagocytosis, destroys tissues and alveolar bone, and affects complement-related channels, while transducing the occurrence of a pro-inflammatory signal cascade.

Inflammation is the body’s biological response to various cellular and tissue damage caused by biological, chemical, and physical factors. If the activity of stimulators and the mechanisms for the proper development of inflammation are disregulated, the body can still be signaled by health hazards and transition from a state of acute to chronic inflammation.^17^ However, Emery et al^14^ and Riviere et al^52^ suggested that a strong statistical association exists between the presence of stimulators and chronic inflammation, which means the body will not undergo progressive inflammation without a stimulator. *P. gingivalis* and its LPS were found at higher frequency in autopsied brain tissues of patients who died of AD; however, they were not found in normal human brain tissues.^26^ This can be conclusive evidence of a connection between these two diseases. The bacterial endotoxin LPS is a major inducer of inflammation causing chronic neuroinflammation, Aβ accumulation/deposition, and cognitive impairment.^37^ *P. gingivalis* infection may be associated with exacerbation of AD pathology, rather than affecting the onset of AD.

The immune invasion strategy of *P. gingivalis* is not only of great significance in PD but is also related to systemic diseases, because *P. gingivalis* and its virulence factors can penetrate many organs. When doing this, *P. gingivalis* and/or its products, as well as any inflammatory mediators produced in the blood, can reach other, distant organs in the body. With routine dental surgery, including tooth extraction, gingivectomy, flap surgery, and even flossing and brushing, oral cavity bacteria enter the systemic circulation.^13,51^ *P. gingivalis* and its LPS are potent initiators of inflammatory signaling in the periphery and in the brain, which have direct and early effects on memory.

PD may be an important source of systemic inflammatory molecules, exacerbating the inflammatory response in organs. There are two different possible pathways by which pathogens can enter the brain from the mouth. The first is through the systemic circulation. Inflammatory processes in periodontal tissue can reach the brain through the circulatory system and circumventricular organs with pro-inflammatory cytokines, but bacterial contact with brain tissue does not occur. However, once bacteria are in the brain, pro-inflammatory molecules can directly increase the expression levels of a panel of pro-inflammatory cytokines or indirectly activate glial cells by modulating the secretion of additional pro-inflammatory cytokines.^10^ The second pathway is neural. Periodontal bacteria or bacterial molecules can enter the central nervous system either through the blood stream (hematogenous routes) or via peripheral nerves.^10,24^ On the other hand, the intramural periarterial drainage pathway (IPAD), is only involved in the removal of solutes from the brain parenchyma along the basement membrane of arterial smooth muscle cells.^1,38^ This may be a new pathway for pathogens to enter the brain. Clearance of LPS by the IPAD and glymphatic system likely employs the same pathway that Aβ uses to exit the brain.^1^ LRP1 is the primary transporter of Aβ efflux from the BBB, while RAGE can transport Aβ from the circulation into the brain (Fig 1).^32^ Therefore, activation of LRP1 and inhibition of RAGE can reduce Aβ entry into the brain.

On the other hand, *P. gingivalis* is also equipped with two types of gingipains, Kgp and arginine-specific Rgp, which are distinguished by the specificity of their cleavage sites. Gingipains are large proteins (180 kD) encoded by kgp or rgpA genes, containing a signal peptide, N-terminal pro-fragment, Kgp or Rgp catalytic domain, and a C-terminal multi-domain hemagglutinin/adhesin (HA1-HA4) region.^33^ Kgp and Rgp are cysteine proteases attached to the surface of bacteria or secreted into the environment.^45^ *P. gingivalis* is the only bacterium that produces gingipains, an endopeptidase that is responsible for 85% of its proteolytic activity. Gingipains are known to play an important role in the progression of PD by causing inflammation and destruction of periodontal tissue; they play an important role in bacterial-mediated host cell responses and subsequent intracellular signaling in infected cells.^41^

In in-vitro culture, *P. gingivalis* causes AD-like neurodegeneration in neurons and continues to express active gingipains by the following mechanism: *P. gingivalis* can invade and survive in neurons and produce intra-neuronal gingipains with proteolytic activity, resulting in AD-related neurodegeneration.^12,45^

In order to evaluate whether the capsulated strains of *P. gingivalis* display different capacities of inducing cognitive impairment, Díaz-Zúñiga et al^9^ quantified Rgp and Kgp gingipain genes in hippocampal tissue by qRT-PCR. They found that both genes were detected in the hippocampus, but no differences were found among the different serotypes. Olsen et al^45^ demonstrated the presence of gingipains in over 90% of postmortem AD brains, with gingipains localised to the cytoplasm of neurons. Although different serotypes of *P. gingivalis* can enter the brain, its particular capsular types play a central role in the onset of chronic inflammation and cognitive impairment induced by short-term oral infection.

Oral infection of wild-type mice with *P. gingivalis* resulted in the production of gingipains as well as p-Tau in neurons, which were not seen in uninfected mice.^42^ Furthermore, *P. gingivalis* has been shown to secrete other components associated with periodontitis that may also affect cognitive function.

## Genetic Factors Linking Peridontal Disease to Alzheimer’s Disease

Some studies have analysed the relationship between the *P. gingivalis*-host interactome and the genes identified in genome-wide association studies (GWAS) to determine the way genes integrate PD and AD. They studied conditions using GWASdb data (p < 1E-03) and in some cases from the NCBI/EBI GWAS database (p < 1E-05).^6^ Harding et al^20^ analysed the relationship between the *P. gingivalis*-host interactome and the genes identified in a genome-wide association study (GWAS) to determine how the genes link PD to AD. With periodontitis gene expression or *P. gingivalis,* microarrays were compared to microarray datasets from the AD hippocampus and/or from carotid artery plaques; the results demonstrated that host genes of the *P. gingivalis* interactome were statistically significantly enriched in GWAS genes related to cognitive impairment, AD, and dementia. The *P. gingivalis*-host interactome was also enriched in GWAS genes according to the more stringent NCBI-EBI database for AD, atherosclerosis, and T2DM.^20^ The deregulated genes in periodontitis tissue or *P. gingivalis*-infected macrophages also matched those in the AD hippocampus or atherosclerotic plaques.^2^ Together, these data suggested important gene/environment interactions between* P. gingivalis* and susceptibility genes or changes in gene expression under conditions where PD is a concomitant factor. In the stimulation and maintenance of inflammation, epigenetic pathways are of particular interest due to their upstream regulation. Epigenetic modifications result in chemical changes in DNA and related proteins that lead to chromatin remodeling and inactivation or activation of gene transcription. These changes promote the development and maintenance of cancer, autoimmune and inflammatory diseases, including PD and AD.^11,47^ Therefore, understanding of the modification of epigenetic mechanisms may help to gain insight into the key regulatory pathways of genes involved in the maintenance of chronic inflammation. Thus, the role of DNA and histone modifications, as major epigenetic regulators in periodontitis, have been described, where gene expression is influenced by DNA methylation.^36^ It has also been demonstrated that the chronic inflammation of periodontitis may be related to abnormal DNA methylation in gingival tissue. In AD, it has been found that the epigenetic mechanism is dysregulated during the progression of the disease, especially at an early stage.^63^ In addition, recent methylome-wide association studies (MWAS) in humans supports the notion that abnormal DNA methylation is associated with AD.^35^

A current hypothesis suggests that *P. gingivalis* infection plays a role in AD pathogenesis by secreting gingipains to promote neuronal damage. Dominy et al^12^ found that immune reactivity (IR) to gingipains was significantly higher in AD brains than in brains of non-AD control individuals. In addition, *P. gingivalis* DNA was identified in AD brains and the cerebrospinal fluid (CSF) of living subjects diagnosed with probable AD, suggesting that CSF *P. gingivalis* DNA may serve as a differential marker to diagnose AD. Genetic polymorphisms in genes of the innate immune system in essential immune pathways may lead to defective clearance of *P. gingivalis* and gingipains from the brain, resulting in low-level infection as well as chronic and neuroinflammation in susceptible individuals.

A recent study found *P. gingivalis* genomic DNA and LPS at high frequencies in autopsied brain tissues of patients who died of AD; however, they were not found in normal human brain tissues,^51^ which was significant evidence that *P. gingivalis* is a vital pathway in AD.

In recent studies on AD, apolipoprotein E (ApoE) has been found to be a lipid transporter in the peripheral and central nervous systems.^31^ ApoE lipoprotein particles bind to various cell surface receptors, maintain cell membrane homeostasis and repairing damage in the brain. Taking into account the prevalence and relative risk, the apolipoprotein gene allele 4 (ApoE ε4) is the strongest genetic risk factor for late-onset AD.^59^ This distinction is important, given that ApoE ε4 acts as a susceptibility gene interacting with environmental risk factors, such as a sub-gingival pathobiome responsible for PD, which may enhance its biological function in favour of AD.^4,5,59,69^ ApoE ε4 promotes the pathogenesis of AD by regulating multiple pathways, including but not limited to metabolism, Aβ peptide aggregation and toxicity, Tau disease, lipid transport, synaptic plasticity, vascular integrity, mitochondrial function, and nerve inflammation.^4^

Above all, ApoE ε4 is the only major susceptibility gene for late-onset AD. However, the genetic architecture of late-onset AD is far from fully understood. Zhao et al^69^ described the biochemical and biological characterisation of ApoE and ApoE-receptors in the central nervous system. They also discussed the mechanisms and evidence for the different roles of ApoE receptors and ApoE isoforms in AD pathogenesis, with particular emphasis on the preclinical and clinical investigations related to Aβ pathology.

Rokad et al^53^ and Singhrao et al^56^ first provided a proof-of-concept that *P. gingivalis* following bacteremia can translocate from its oral niche to the brain. Furthermore, peripheral inflammation was found to negatively affect BBB integrity. In addition, *P. gingivalis* entry was directly related to the innate immune responses that a) had an impact on intracerebral inflammation in the form of ROS and b) complement activation. These are important findings that outweigh the inability to assess Aβ pathology in this periodontitis-AD model.

In the case of ApoE-/- experimental *P. gingivalis* oral infection in ApoE mice, the host releases large amount of IL-10 in the serum. However, Poole et al^50^ found that the bacteria themselves still spread to the brain and encounter microglia, which become activated as a result. It is noteworthy that *P. gingivalis* was detected in the brain microglia and hippocampal capillaries of ApoE-/- mice from the *P. gingivalis* infection group, which means *P. gingivalis* invasion of ApoE-/- induced complement activation in mouse brains.^24^ Poole et al^50^ also showed that *P. gingivalis* was able to enter the ApoE-/- brain and contribute to the development of AD inflammatory pathology through mechanisms involving cytokines, acute phase proteins, and the complement cascade, in which neurons are attacked.

Likewise, findings by Olsen et al^46^ on mono-infection ApoE-/- mice infected with *P. gingivalis* suggested that increased carbonyl protein-related oxidative stress is present in their cerebral microvasculature following induction of experimental periodontitis and post-atherosclerotic lesion appearance. Exacerbation of vascular pathological changes may be a plausible factor in reducing adequate oxygen supply to the brain.

All of these studies provided feasible ideas to test the genetic factors of *P. gingivalis* infection in AD.

## Activation of Glial Cells and Leptomeningeal Cells by *P. gingivalis* in AD

Microglia make up 10% of the total number of brain cells. Astrocytes and microglia are CNS-resident immune cells that can perform both detrimental (activation of neurotoxic immune responses) and beneficial (reduction of immune responses) functions. Peripheral infections could activate already-primed microglia within the CNS, which contributes to the development of neurodegeneration in AD.^55,62^

Neuroinflammation, including microglial activation, astrocyte and neuronal involvement, is an inflammatory response in the CNS in response to injury or infection and accumulates in glial cells. During this process, chemokines, complement, cytokines, pattern-recognition receptors (PRRs), and cellular and molecular immune factors, may activate microglia and astrocytes, which are thought to contribute to the development of neurodegenerative diseases such as AD.^22^

Chronic systemic exposure to *P. gingivalis* LPS has been reported to induce AD-like phenotypes, including those mediated by microglia.^66^ Microglia-mediated neuroinflammation is a key factor in AD pathology, not a consequence of the disease. One possible mechanism for *P. gingivalis* infection in AD is that *P. gingivalis* in the brain promotes chronic neuroinflammation, in which reactive astrocytes and activated microglia actively produce inflammatory mediators, enhancing Aβ production. Accumulation of the latter leads to Tau hyperphosphorylation, which constitutes the histopathological hallmark of AD.^33^ Microglia can “remember” a previous inflammatory challenge and become tolerant or trained to toxins such as LPS. Liu et al^34^ reported that *P. gingivalis*-derived LPS activates microglia to produce pro-inflammatory mediators through toll-like 2 receptors (TLR-2).

As mentioned above, *P. gingivalis* can produce a unique class of cysteine proteases termed gingipains, consisting of Kgp and Rgp, which are major factors involved in bacterially mediated host-cell responses and the subsequent intracellular signaling in infected cells. Therefore, Liu et al^33^ proposed that Kgp and Rgp are involved in the cellular activation of microglia after brain infection. It is thought that Kgp and Rgp may limit microglia-mediated neuroinflammation through proteolytic degradation of pro-inflammatory cytokines. Kgp and Rgp together promote the migration of microglia to the infected site and induce neuroinflammation after infiltrating the brain.^33^

In the in-vivo experiments performed by Liu et al,^34^ CX3CR1+/GFP mice were used to assess the migration of microglia towards the *P. gingivalis* injection site. It has been reported that microglia in the brain express significantly higher levels of CX3CR1 than do macrophages.^23^ In the present study, the majority of CX3CR1-positive cells that accumulated around the sites of injected urethane and atropine (1.7 g/kg and 0.4 mg/kg, respectively) had process-bearing morphologies. Therefore, it is thought that brain-resident microglia can be distinguished from infiltrating macrophages by counting bright CX3CR1-positive cells bearing processes. Furthermore, process-bearing bright CX3CR1-positive cells clustered around the injection site were negative for Ki,^67^ but only a few CX3CR1-positive cells with spindle shapes, probably infiltrating macrophages, were positive for Ki.^67^ These observations suggested that the accumulation of cells around the injection site were primarily associated with cell migration.

In addition, *P. gingivalis* infection also induced microglia to produce pro-inflammatory cytokines and additional Aβ. *P. gingivalis* infection of microglia significantly increased the mRNA expression of pro-inflammatory mediators, including TNF-α, IL-6, and iNOS, but not anti-inflammatory mediators, including IL-10, arginase-1, and IL-4.^10,16,26,34^ These pro-inflammatory mediators play significant roles in Aβ formation. Aβ plaques can eventually be surrounded by glial cells with dysfunctional homeostatic control and thus acquire a pro-inflammatory phenotype that amplifies neuronal damage.

Similarly, recent studies have shown that leptomeningeal cells play an important role in transducing systemic inflammatory signals to the brain-resident microglia that initiate neuroinflammation. Leptomeningeal cells express both TLR-2 and TLR-4, the receptors of *P. gingivalis* LPS, and transmit signals from systemic immune cells to microglia in the brain.^35^ *P. gingivalis* LPS may activate leptomeningeal cells, triggering the direct release of inflammatory molecules into the brain, which activate microglia in the brain. Using an in-vitro mouse model, Liu et al^35^ found that leptomeningeal cells can transmit inflammatory signals from peripheral macrophages to brain-resident microglia in response to stimulation by *P. gingivalis* LPS.

Taken together, *P. gingivalis* LPS and gingipains can contribute to AD by activating microglia and astrocytes, inducing them to release cytokines and influencing the migration of microglia. These factors enhance Aβ production and Tau hyperphosphorylation, which constitute the histopathological hallmarks of AD.

**Fig 2:**
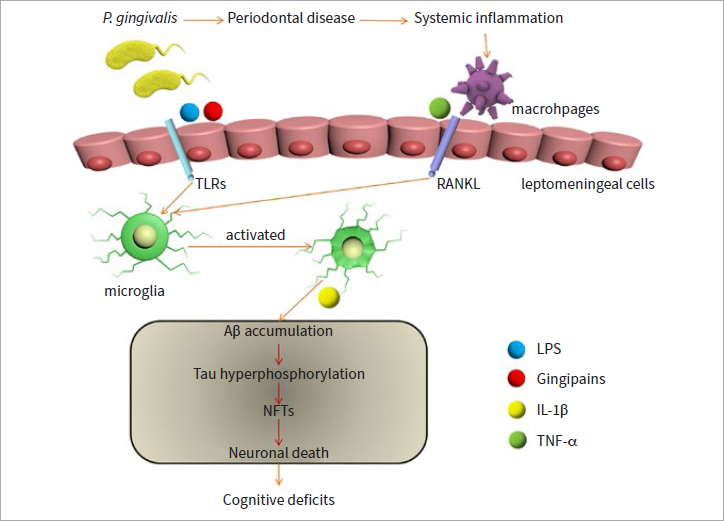
Schematic diagram of periodontal bacterial inflammatory signal transduction through leptomeningeal cells to microglia in the brain. During chronic periodontitis, IL-1β and TNF-α secreted by macrophages and periodontal bacterial components (including LPS and gingipains) activate RANKL and TLRs located on the surface of the leptomeningeal cells. These factors in turn activate senescent-type microglia in the brain. After cell activation, senescent-type microglia secrete pro-inflammatory molecules, such as IL-1β, which lead to increased Aβ accumulation. In addition, IL-1β secreted by activated senescent-type microglia also accelerates the formation of tangles through tau hyperphosphorylation. These pathological features of AD may impair neuronal function and promote cognitive deficits.

In this cascade, leptomeningeal cells may play an important role in transducing systemic inflammatory signals to the brain-resident microglia (Fig 2).

## Immune Responses to Oral *P. gingivalis* in AD

As mentioned above, microglia and astrocytes play an eminent role in the immune system of the CNS. The brain is less dependent on recruiting peripheral adaptaive immune-surveillance cells and more dependent on resident CNS cells to identify and respond to invading pathogens. However, outside the BBB, in periodontitis and related conditions, dysbiosis as a cause of inflammation is achieved by manipulating the host’s adaptive immune system, including cellular immunological responses.^8,46^ Strong peripheral inflammation caused by bacterial infection leads to peripheral leukocyte (macrophages, T-cells, and neutrophils) infiltration of the CNS, which shares several functional features with microglia. Leukocytes express TLRs and can be activated by abnormal proteins or pathogen-associated molecular patterns (PAMPs; pathogens replicate and release their component molecules).^10,18,62^ Therefore, the acute neuroinflammatory response is beneficial, repairing damaged areas of the brain and helping restore brain homeostasis. However, if the inflammatory response is uncontrolled and chronic, long-term activation is characterised by the microglia releasing pro-inflammatory mediators, increased oxidative and nitrosative stress; thus, the inflammatory cycle continues and contributes to neurodegenerative diseases such as AD.^10,25^ When the threshold concentration of bacteria and/or its immunogenic components is reached, the classical innate immune signaling pathway is initiated via TLR-2 and TLR-4 mechanisms, inevitably leading to cytokine release by microglia.

Chronic release of cytokines ultimately alters BBB permeability and reduces the outflow of Aβ from the CNS into the systemic circulation. This means Aβ increases in brain tissues and becomes a pathological change in terms of AD. In this pathway, *P. gingivalis* LPS is an important contributor, because pattern recognition receptors (PRR), such as TLRs, are expressed by glial cells and PAMPs can trigger antibacterial responses in microorganisms. *P. gingivalis* LPS stimulates TLR-2 or -4, as well as CD14, and sends a signal to the nucleus via the MyD88 pathway, triggering a cascade of events involving increased expression of pro-inflammatory cytokines.^10,25^

By preventing immune cells from entering the brain, *P. gingivalis* can affect BBB permeability and block local IFN-γ responses in AD. The lack of adaptive immune cells in AD neuropathology means that *P. gingivalis* infection in the brain may lead to impaired clearance of insoluble amyloids and induce immunosuppression.

*P. gingivalis* has many surface membranes, enzymes and capsular proteins that inhibit neutrophil recruitment of chemokines (signaling proteins or a cell-secreted family of small cytokines) by cleaving CD14 and immune-cell receptors (receptor activator of NF-κb ligand, RANKL), eroding cells, or inducing subversive cross-talk signaling between TLR-2 and other innate immune receptors, such as the C5aR anaphylatoxin receptor.^44^

The level of periodontal inflammation and immune responses might be used to assess risk factors for developing systemic diseases; this implies that identifying *P. gingivalis* infection might aid in the diagnosis of periodontitis-associated systemic diseases. Hirai et al^24^ aimed to identify *P. gingivalis* antigens that are specifically recognised by serum immunoglobulin G (IgG), which is present in large amounts in periodontitis patients vs healthy individuals. Using enzyme-linked immunosorbent assays (ELISA) to measure the serum IgG-antibody level against periodontal pathogens, those authors determined humoral immunological responses against periodontal pathogens. Periodontal bacterial infections are known to induce humoral immunological responses and increase serum IgG-antibody levels against pathogens. As their results showed, serum IgG antibody levels against* P. gingivalis* indicate the level of periodontal inflammation; these levels are appropriate markers for screening patients with periodontitis. Furthermore, they found that periodontitis patients had the highest recombinant Rgp among the antigens expressed in *P. gingivalis*.

However, serum IgG levels for periodontal pathogens are not tested during a clinical examination. One of the reasons is that the use of antigens in the media from sonicated preparations of cultured pathogens can lead to nonspecific immune responses with IgG antibodies. Another reason is the difficulty in standardising the bacterial antigens in ELISA. This assay is a potentially useful lab method for finding a new approach to studying *P. gingivalis* infection in AD.^24^

*P. gingivalis* alone is not responsible for periodontal disease, systemic diseases or dementia, nor are periodontitis and its related systemic diseases the effect of a single bacterial species. Hence, other factors influencing PD or AD should be considered.

## Three-way Relationships among *P. gingivalis* Infection, AD, and Age

The incidence of AD increases exponentially with age, from 3% in the 65- to 74-year-old age group to nearly 50% in the 85-year-plus age group. Furthermore, nutrition and its subsequent impact on oral health are often overlooked and should be a priority. Although oral health may be less important compared with the management of other diseases related to old age, it is clear that poor oral health may cause or aggravate other diseases. It follows that the currently increasing numbers of elderly with inadequate dental care are entering the high-risk group for AD. Comparing times of onset, PD appears after the age of 30 years, while late-onset AD begins at over 80 years. Therefore, as shown by Singhrao et al,^58^ established chronic periodontal pathogens (such as *P. gingivalis*) have enough time to use blood-borne pathways to enter the brain. A retrospective study conducted by Chen et al^6^ found a strong link between chronic PD (exposure of around 10 years) and AD, and this correlated with a prospective laboratory-based study in which circulating antibodies of oral bacteria were associated with a cognitive deficit 10 years later. This suggested that the 10-year lag in chronic periodontitis is plausible as a risk factor for sporadic AD. Why is there a long lag period? It is possible that during aging, initial weakening of the protective BBB occurs, which makes it easier for bacteria to enter the brain. Alternatively, unlike the oral cavity, which contains a range of different bacterial systems, such as supra- and subgingival plaque, and develops a chronic infection only a few weeks later, the healthy brain may be slow to respond to nominally virulent (seronegative) *P. gingivalis* in younger human hosts because they have healthy immune systems.^59^ It is necessary to emphasise protective barriers such as the BBB which keep the CNS sequestered and protect against abnormal immune challenges (entry of toxins and associated cells that detoxify foreign proteins) early in life.

Ding et al^10^ found that the escape latency between day-1 infected *P. gingivalis* mice and control mice did not differ statistically significantly, and *P. gingivalis* infection had no statistically significant effect on cognition in young mice. However, on days 2, 3, and 4, the escape latency of the middle-aged *P. gingivalis* infected mice differed statistically significantly from the control group. *P. gingivalis* infection obstructed the spatial memory and learning abilities of the middle-aged mice. In addition, the mean mRNA expression levels of TNF-α in the brain tissue of middle-aged *P. gingivalis*-infected mice were higher compared with controls. However, no statistically significant effect on TNF-α mRNA levels was observed in the young mouse group. Likewise, IL-6 and IL-1β mRNA levels were only increased in middle-aged *P. gingivalis*-infected mice, but not in young mice.^10^ Overall, the levels of all of the three pro-inflammatory cytokines were statistically significantly higher in middle-aged *P. gingivalis*-infected mice than in control mice. In contrast, the concentration of IL-6, IL-1β, and TNF-α did not differ between the two groups of young mice, suggesting that *P. gingivalis* infection may increase the expression of pro-inflammatory cytokines IL-6, IL-1β, and TNF-α in the brain tissues, which promotes neuroinflammation of middle-aged mice.^10,16,25,33^ The expression of these cytokines showed differences between middle-aged and young mice. Chronic systemic inflammatory processes promoted the transformation of microglia and astrocytes into anti-inflammatory cell types in young rats, whereas a pro-inflammatory cell phenotype was detected in middle-aged rats. Furthermore, age strongly affects the barrier functions of leptomeningeal cells through age-dependent differential microglial responses during chronic systemic inflammation.^67^

Aging-induced chronic inflammation may result from these differences between young and middle-aged mice. This puts additional stress on the brain neurons of older mice, making them more vulnerable to infection. Therefore, it is necessary to consider the effect of aging on neuroinflammation.

## Conclusion and Outlook

PD, caused by *P. gingivalis*, is a chronic persistent inflammatory response triggered by subgingival bacterial biofilm, which results in chronic inflammatory destruction of the gingival connective tissue attached to the root surface, cementum, and adjacent alveolar bone. The immune invasion strategy of *P. gingivalis* is not only important in PD, but is also implicated in other systemic diseases when the bacterium and its virulence factors enter systemic organs.

Neuroinflammation is so pivotal that scientists question whether AD is an infectious disease. In this complex interaction of different players, microglia appear to be important in the host defense against invasion by the key periodontal pathogen *P. gingivalis*.

Proteostasis (Tau and/or Aβ plaques) is a central component of pathology in AD. AD is characterised by the accumulation of intracellular neurofibrillary tangles, which are composed of extracellular deposits of the microtubule binding protein Tau and Aβ fibrils assembled into pairs of spirals and straight filaments. *P. gingivalis* may co-evolve with the host’s immune defense development strategies to overcome the host’s protective barriers and modulate the host’s defense systems to its own advantage. This is attributed to the ability of *P. gingivalis* to endure inflammation and exploit it for its own survival and maintenance.

Future studies are needed to clarify the exact pathway and mechanisms of *P. gingivalis* infections and AD, and to identify effective treatments. Early diagnosis and treatment of PD is crucial to retard disease progression.

## References

[ref1] Albargothy NJ, Johnston DA, MacGregor-Sharp M, Weller RO, Verma A, Hawkes CA (2018). Convective influx/glymphatic system: tracers injected into the CSF enter and leave the brain along separate periarterial basement membrane pathways. Acta Neuropathol.

[ref2] Bale BF, Doneen AL, Vigerust DJ (2017). High-risk periodontal pathogens contribute to the pathogenesis of atherosclerosis. Postgrad Med J.

[ref3] Borsa L, Dubois M, Sacco G, Lupi L (2021). Analysis the link between periodontal diseases and Alzheimer’s Disease: a systematic review. Int J Environ Res Public Health.

[ref4] Brabec JL, Lara MK, Tyler AL, Mahoney JM (2021). System-level analysis of Alzheimer’s Disease prioritizes candidate genes for neurodegeneration. Front Genet.

[ref5] Carter CJ, France J, Crean S, Singhrao SK (2017). The *Porphyromonas gingivalis*/host interactome shows enrichment in GWASdb genes related to Alzheimer’s Disease, diabetes and cardiovascular diseases. Front Aging Neurosci.

[ref6] Chen CK, Wu YT, Chang YC (2017). Association between chronic periodontitis and the risk of Alzheimer’s disease: a retrospective, population-based, matched cohort study. Alzheimers Res Ther.

[ref7] Darveau RP, Curtis MA (2021). Oral biofilms revisited: A novel host tissue of bacteriological origin. Periodontol 2000.

[ref8] Dekita M, Wu Z, Ni JJ, Zhang XW, Liu YC, Yan X (2017). Cathepsin S is involved in Th17 differentiation through the upregulation of IL-6 by activating PAR-2 after systemic exposure to lipopolysaccharide from *Porphyromonas gingivalis*. Front Pharmacol.

[ref9] Díaz-Zúñiga J, More J, Melgar-Rodríguez S, Jiménez-Unión M, Villalobos-Orchard F, Konradi A (2020). Alzheimer’s Disease-like pathology triggered by* Porphyromonas gingivalis* in wild type rats is serotype dependent. Front Immunol.

[ref10] Ding Y, Ren JY, Yu HQ, Yu WX, Zhou YM (2018). *Porphyromonas gingivalis*, a periodontitis causing bacterium, induces memory impairment and age-dependent neuroinflammation in mice. Immun Ageing.

[ref11] Diomede F, Thangavelu SR, Merciaro I, D’Orazio M, Bramanti P, Mazzon E (2017). *Porphyromonas gingivalis* lipopolysaccharide stimulation in human periodontal ligament stem cells: role of epigenetic modifications to the inflammation. Eur J Histochem.

[ref12] Dominy SS, Lynch C, Ermini F, Benedyk M, Marczyk A, Konradi A (2019). *Porphyromonas gingivalis* in Alzheimer’s disease brains: Evidence for disease causation and treatment with small-molecule inhibitors. Sci Adv.

[ref13] Emery DC, Cerajewska TL, Seong J, Davies M, Paterson A, Allen-Birt SJ (2021). Comparison of blood bacterial communities in periodontal health and periodontal disease. Front Cell Infect Microbiol.

[ref14] Emery DC, Shoemark DK, Batstone TE, Waterfall CM, Coghill JA, Cerajewska TL, Cerajewska TL (2017). 16S rRNA next generation sequencing analysis shows bacteria in Alzheimer’s post-mortem brain. Front Aging Neurosci.

[ref15] Forny-Germano L, Lyra e Silva NM, Batista AF, Brito-Moreira J, Gralle M, Boehnke SE (2014). Alzheimer’s disease-like pathology induced by amyloid-β oligomers in nonhuman primates. J Neurosci.

[ref16] Frost GR, Li YM (2017). The role of astrocytes in amyloid production and Alzheimer’s disease. Open Biol.

[ref17] Fulop T, Witkowski JM, Olivieri F, Larbi A (2018). The integration of inflammaging in age-related diseases. Semin Immunol.

[ref18] Gate D, Saligrama N, Leventhal O, Yang AC, Unger MS, Boehnke SE (2020). Clonally expanded CD8 T cells patrol the cerebrospinal fluid in Alzheimer’s disease. Nature.

[ref19] Gurav AN (2014). Alzheimer’s disease and periodontitis – an elusive link. Rev Assoc Med Bras.

[ref20] Harding A, Robinson S, Crean S, Singhrao SK (2017). Can better management of periodontal disease delay the onset and progression of Alzheimer’s Disease?. J Alzheimers Dis.

[ref21] Hayashi K, Hasegawa Y, Takemoto Y, Cao C, Takeya H, Komohara Y (2019). Continuous intracerebroventricular injection of Porphyromonas gingivalis lipopolysaccharide induces systemic organ dysfunction in a mouse model of Alzheimer’s disease. Exp Gerontol.

[ref22] Heneka MT, Carson MJ, El Khoury J, Landreth GE, Brosseron F, Feinstein DL (2015). Neuroinflammation in Alzheimer’s disease. Lancet Neurol.

[ref23] Hickman SE, El Khoury J (2019). Analysis of the microglial sensome. Methods Mol Biol.

[ref24] Hirai K, Yamaguchi-Tomikawa T, Eguchi T, Maeda H, Takashiba S (2020). Identification and modification of *Porphyromonas gingivalis* cysteine protease, gingipain, ideal for screening periodontitis. Front Immunol.

[ref25] Ilievski V, Zuchowska PK, Green SJ, Toth PT, Ragozzino ME, Le K (2018). Chronic oral application of a periodontal pathogen results in brain inflammation, neurodegeneration and amyloid beta production in wild type mice. PLoS One.

[ref26] Ishida N, Ishihara Y, Ishida K, Tada H, Funaki-Kato Y, Hagiwara M (2017). Periodontitis induced by bacterial infection exacerbates features of Alzheimer’s disease in transgenic mice. NPJ Aging Mech Dis.

[ref27] Joseph S, Curtis MA (2021). Microbial transitions from health to disease. Periodontol 2000.

[ref28] Jungbauer G, Stähli A, Zhu X, Auber Alberi L, Sculean A, Eick S (2022). Periodontal microorganisms and Alzheimer disease – A causative relationship?. Periodontol 2000.

[ref29] Kamer AR, Fortea JO, Videla S, Mayoral A, Janal M, Carmona-Iragui M (2016). Periodontal disease’s contribution to Alzheimer’s disease progression in Down syndrome. Alzheimers Dement (Amst).

[ref30] Ke XJ, Lei L, Li H, Li HX, Yan FH (2016). Manipulation of necroptosis by *Porphyromonas gingivalis* in periodontitis development. Mol Immunol.

[ref31] Leon M, Sawmiller D, Giunta B, Tan J (2018). Therapeutic approach targeting apolipoprotein E binding region and low-density lipoprotein receptor for Alzheimer’s disease. Neuroimmunol Neuroinflamm.

[ref32] Liu J, Wang Y, Guo J, Sun J, Sun Q (2020). Salvianolic Acid B improves cognitive impairment by inhibiting neuroinflammation and decreasing Aβ level in *Porphyromonas gingivalis* -infected mice. Aging (Albany NY).

[ref33] Liu YC, Wu Z, Nakanishi Y, Ni JJ, Hayashi Y, Takayama F, Takayama F (2018). Author Correction: Infection of microglia with Porphyromonas gingivalis promotes cell migration and an inflammatory response through the gingipain-mediated activation of protease-activated receptor-2 in mice. Sci Rep.

[ref34] Liu YC, Wu Z, Zhang XW, Ni JJ, Yu WX, Zhou Y (2013). Leptomeningeal cells transduce peripheral macrophages inflammatory signal to microglia in reponse to Porphyromonas gingivalis LPS. Mediators Inflamm.

[ref35] Liu YY, Wang MH, Marcora EM, Zhang B, Goate AM (2019). Promoter DNA hypermethylation – implications for Alzheimer’s disease. Neurosci Lett.

[ref36] Martins MD, Jiao Y, Larsson L, Almeida LO, Garaicoa-Pazmino C, Le JM (2016). Epigenetic Modifications of Histones in Periodontal Disease. J Dent Res.

[ref37] Mihaylova A, Doncheva N, Zlatanova H, Delev D, Ivanovska M, Koeva Y (2021). Dopaminergic agonist pramipexole improves memory and increases IL-10 production in LPS-challenged rats. Iran J Basic Med Sci.

[ref38] Morris AW, Sharp MM, Albargothy NJ, Fernandes R, Hawkes CA, Verma A, Weller RO, Carare RO (2016). Vascular basement membranes as pathways for the passage of fluid into and out of the brain. Acta Neuropathol.

[ref39] Mysak J, Podzimek S, Sommerova P, Lyuya-Mi Y, Bartova J, Janatova T, Janatova T (2014). *Porphyromonas gingivalis*: major periodontopathic pathogen overview. J Immunol Res.

[ref40] Narasaki CT, Toman R (2012). Lipopolysaccharide of *Coxiella burnetii*. Adv Exp Med Biol.

[ref41] Nemoto TK, Ohara Nemoto Y (2021). Dipeptidyl-peptidases: Key enzymes producing entry forms of extracellular proteins in asaccharolytic periodontopathic bacterium *Porphyromonas gingivalis*. Mol Oral Microbiol.

[ref42] Nobre Dos Santos-Lima EK, Araújo Paiva Andrade Cardoso K, Mares de Miranda P, Cirino de Carvalho-Filho P, Passos Rocha T, Ferreira de Moura-Costa L (2020). Novel synthetic peptide derived from *Porphyromonas gingivalis* Lys-gingipain detects IgG-mediated host response in periodontitis. Anaerobe.

[ref43] Ohlrich EJ, Cullinan MP, Seymour GJ (2009). The immunopathogenesis of periodontal disease. Aust Dent J.

[ref44] Olsen I, Hajishengallis G (2016). Major neutrophil functions subverted by *Porphyromonas gingivalis*. J Oral Microbiol.

[ref45] Olsen I, Singhrao SK (2020). Interaction between genetic factors, *Porphyromonas gingivalis* and microglia to promote Alzheimer’s disease. J Oral Microbiol.

[ref46] Olsen I, Taubman MA, Singhrao SK (2016). *Porphyromonas gingivalis* suppresses adaptive immunity in periodontitis, atherosclerosis, and Alzheimer’s disease. J Oral Microbiol.

[ref47] Olsen I, Yilmaz Ö (2019). Possible role of *Porphyromonas gingivalis* in orodigestive cancers. J Oral Microbiol.

[ref48] Percy ME, Lukiw WJ (2020). Is heart disease a risk factor for low dementia test battery scores in older persons with Down syndrome? Exploratory, pilot study, and commentary. Int J Dev Disabil.

[ref49] Poole S, Singhrao SK, Chukkapalli S, Rivera M, Velsko I, Kesavalu L (2015). Active invasion of *Porphyromonas gingivalis* and infection-induced complement activation in ApoE-/- mice brains. J Alzheimers Dis.

[ref50] Poole S, Singhrao SK, Kesavalu L, Curtis MA, Crean S (2013). Determining the presence of periodontopathic virulence factors in short-term postmortem Alzheimer’s disease brain tissue. J Alzheimers Dis.

[ref51] Rezazadeh F, Azad A, Khorami A, Modaresi F, Rezaie Z (2020). Evaluation of antibiotic resistance pattern in dental bacteremia detected by multiplex PCR technique. Biomed Res Int.

[ref52] Riviere GR, Riviere KH, Smith KS (2002). Molecular and immunological evidence of oral Treponema in the human brain and their association with Alzheimer’s disease. Oral Microbiol Immunol.

[ref53] Rokad F, Moseley R, Hardy RS, Chukkapalli S, Crean S, Kesavalu L (2017). Cerebral oxidative stress and microvasculature defects in TNF-α expressing transgenic and *Porphyromonas gingivalis* -Infected ApoE-/- Mice. J Alzheimers Dis.

[ref54] Sedghi L, DiMassa V, Harrington A, Lynch SV, Kapila YL (2021). The oral microbiome: role of key organisms and complex networks in oral health and disease. Periodontol 2000.

[ref55] Selles MC, Oliveira MM, Ferreira ST (2018). Brain inflammation connects cognitive and non-cognitive symptoms in Alzheimer’s Disease. J Alzheimers Dis.

[ref56] Singhrao SK, Chukkapalli S, Poole S, Velsko I, Crean SJ (2017). Chronic *Porphyromonas gingivalis* infection accelerates the occurrence of age-related granules in ApoE-/- mice brains. J Oral Microbiol.

[ref57] Singhrao SK, Harding A, Poole S, Kesavalu L, Crean S (2015). *Porphyromonas gingivalis* periodontal infection and its putative links with Alzheimer’s Disease. Mediators Inflamm.

[ref58] Singhrao SK, Harding A, Simmons T, Robinson S, Kesavalu L (2014). Oral inflammation, tooth loss, risk factors, and association with progression of Alzheimer’s disease J Alzheimers Dis.

[ref59] Singhrao SK, Olsen I (2019). Assessing the role of *Porphyromonas gingivalis* in periodontitis to determine a causative relationship with Alzheimer’s disease. J Oral Microbiol.

[ref60] Sochocka M, Zwolińska K, Leszek J (2017). The infectious etiology of Alzheimer’s Disease. Curr Neuropharmacol.

[ref61] Solleiro-Villavicencio H, Rivas-Arancibia S (2018). Effect of chronic oxidative stress on neuroinflammatory response mediated by CD4+T cells in neurodegenerative diseases. Front Cell Neurosci.

[ref62] Spagnuolo C, Moccia S, Russo GL (2018). Anti-inflammatory effects of flavonoids in neurodegenerative disorders. Eur J Med Chem.

[ref63] Stoccoro A, Coppedè F (2018). Role of epigenetics in Alzheimer’s disease pathogenesis. Neurodegener Dis Manag.

[ref64] Teles F, Wang Y, Hajishengallis G, Hasturk H, Marchesan JT (2021). Impact of systemic factors in shaping the periodontal microbiome. Periodontol 2000.

[ref65] Wade WG (2021). Resilience of the oral microbiome. Periodontol 2000.

[ref66] Wu Z, Ni JJ, Liu YC, Teeling JL, Takayama F, Collcutt A (2017). Cathepsin B plays a critical role in inducing Alzheimer’s disease-like phenotypes following chronic systemic exposure to lipopolysaccharide from Porphyromonas gingivalis in mice. Brain Behav Immun.

[ref67] Wu Z, Tokuda Y, Zhang XW, Nakanishi H (2008). Age-dependent responses of glial cells and leptomeninges during systemic inflammation. Neurobiol Dis.

[ref68] Xu W, Zhou W, Wang H, Liang S (2020). Roles of *Porphyromonas gingivalis* and its virulence factors in periodontitis. Adv Protein Chem Struct Biol.

[ref69] Zhao N, Liu CC, Qiao WH, Bu GJ (2018). Apolipoprotein E, receptors, and modulation of Alzheimer’s Disease. Biol Psychiatry.

